# Origin of Bacteriochlorophyll *a* and the Early Diversification of Photosynthesis

**DOI:** 10.1371/journal.pone.0151250

**Published:** 2016-03-08

**Authors:** Tanai Cardona

**Affiliations:** Department of Life Sciences, Imperial College London, London, United Kingdom; National Research Council of Italy, ITALY

## Abstract

Photosynthesis originated in the domain Bacteria billions of years ago; however, the identity of the last common ancestor to all phototrophic bacteria remains undetermined and speculative. Here I present the evolution of BchF or 3-vinyl-bacteriochlorophyll hydratase, an enzyme exclusively found in bacteria capable of synthetizing bacteriochlorophyll *a*. I show that BchF exists in two forms originating from an early divergence, one found in the phylum Chlorobi, including its paralogue BchV, and a second form that was ancestral to the enzyme found in the remaining anoxygenic phototrophic bacteria. The phylogeny of BchF is consistent with bacteriochlorophyll *a* evolving in an ancestral phototrophic bacterium that lived before the radiation event that gave rise to the phylum Chloroflexi, Chlorobi, Acidobacteria, Proteobacteria, and Gemmatimonadetes, but only after the divergence of Type I and Type II reaction centers. Consequently, it is suggested that the lack of phototrophy in many groups of extant bacteria is a derived trait.

## Introduction

Currently there are seven major phyla of bacteria known to contain strains with photochemical reaction centers. These are Cyanobacteria, Proteobacteria, Firmicutes, Chloroflexi, Chlorobi, Acidobacteria, and Gemmatimonadetes [1]. In addition, a recent study suggested that a few strains in the phylum Actionabecteria may retain what appears to be a vestigial chlorophyll synthesis pathway [2]. From these groups, only Proteobacteria, Chloroflexi, Chlorobi, Acidobacteria, and Gemmatimonadetes are capable of synthetizing bacteriochlorophyll *a*.

Bacteriochlorophyll *a* is synthetized from the precursor, chlorophyllide *a* [3]. The double bond between the C7 and C8 carbons of chlorophyllide *a* is reduced by chlorophyllide *a* oxidoreductase, an enzyme made of three subunits, BchX, BchY, and BchZ, or BchXYZ. This step is followed by the hydration of the C3-vinyl group of the bacteriochlorophyllide *a* precursor to form a hydroxyethil group. The reaction is catalyzed by BchF, also known as 3-vinyl-bacteriochlorophyll hydratase, which is exclusively found in those groups of bacteria that can synthetize bacteriochlorophyll *a* [4]. Subsequently, the hydroxyl group is dehydrogenated to form a keto group before the addition of the phytyl tail; these reactions are catalyzed by BchC and BchG, respectively. In the phylum Cyanobacteria, the BchXYZ, BchF, and BchC enzymes are absent; while in Heliobacteria―the phototrophic Firmicutes―BchXYZ catalyzes the direct formation of bacteriochlorophyll *g* from 8-vinyl-chlorophyllide *a*, but lack BchF and BchC [5].

Proteobacteria, Acidobacteria, Chloroflexi, and Gemmatimonadetes carry a single copy of the BchF enzyme; however, phototrophic Chlorobi usually carry two or three paralogues of BchF, with a second form named BchV [4]. The different versions of BchF in Chlorobi are thought to aid in the synthesis of enantiomeric forms of bacteriochlorophyll *c*, *d*, and *e* that are characteristic of this phylum [6]. BchF is about 170 amino acid-long and is predicted to have four transmembrane helices, suggesting that the later steps of bacteriochlorophyll *a* synthesis occur within the membrane. Currently, the crystal structure of BchF has not been resolved.

The evolution of the chlorophyll and bacteriochlorophyll synthesis pathway has been debated at length before, see for example [4, 7–9]. Most notably, to find out in which group of bacteria photosynthesis originated. In here, I present for the first time a phylogenetic analysis of the BchF enzyme and discuss the implications this has on the evolution of photosynthesis. It is worth noting that because bacteriochlorophyll *a* cannot be synthetized without BchF and because BchF is only and exclusively found in those bacteria that make bacteriochlorophyll *a*; the phylogeny of this enzyme is, in consequence, the most direct piece of evidence for the origin of phototrophy based on bacteriochlorophyll *a*.

## Results

**Fig 1:**
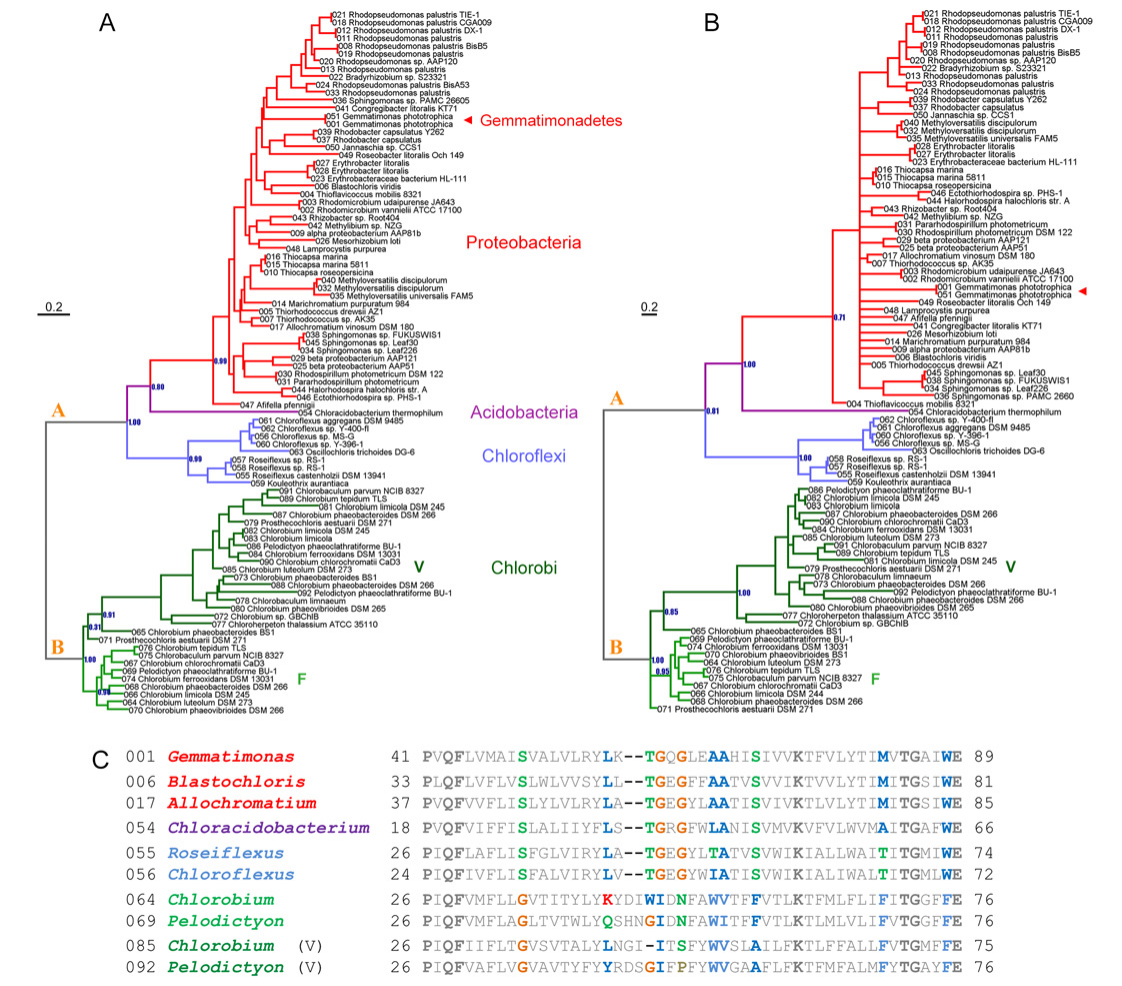
Phylogenetic trees of the BchF protein. **A** shows a Maximum Likelihood tree and **B** shows a tree calculated using Bayesian Inference. The scale bar represents the average number of substitutions per site. **C** shows a sequence alignment of a BchF fragment of selected strains representatives of all phyla. In color bold letters I highlighted conserved positions which differentiate the Chlorobi-type from those in the remaining phyla as described in the text. The V in parenthesis denotes the paralogue sequences to BchF usually found in the genomes of phototrophic Chlorobi. The phylogenetic trees and complete sequence alignments are provided in S1 Dataset.

Phylogenetic trees of the BchF protein calculated using Maximum Likelihood and Bayesian Inference are shown in **Fig 1**. Both methods retrieved a similar topology showing a consistent pattern of relations among the different phyla of bacteria. First, BchF exists in two major forms denoted **A** and **B** in **Fig 1.** Form **A** is found in the phyla Chloroflexi, Acidobacteria, Proteobacteria, and Gemmatimonadetes, and form **B** is found in the phylum Chlorobi, which included the isoform, BchV. The differences between these two forms are clearly visible in the sequence alignments, with those present in the phylum Chlorobi showing an insertion of two amino acids between the first and second transmembrane helices in addition to other specific sequence variations, some of which are highlighted in **Fig 1C**. This suggests that early in the evolution of bacteriochlorophyll *a* synthesis two forms of BchF arose: one ancestral to all BchF proteins today only found in the phylum Chlorobi (form **B**), and a second form that gave rise to those present in the remaining phyla (form **A**). This implies that both forms originated from an early gene duplication event in an ancestral phototroph capable of bacteriochlorophyll *a* synthesis or alternatively, as a population of ancestral phototrophic bacteria diverged into two new species. This is independent of whether horizontal gene transfer has occurred at any point in time or not. As a result, the root of the BchF tree should be placed in the branch that separates form **A** from form **B**.

Of particular importance is the position of the BchF from *Chloracidobacterium thermophilum* (Acidobacteria), which appears consistently as the most closely related protein to those present in the phylum Proteobacteria. In other words, the BchF in phototrophic Acidobacteria and Proteobacteria share a more recent common ancestral protein between each other than with the protein forms in the phylum Chloroflexi or Chlorobi. Furthermore, the BchF protein found in strains of the phylum Gemmatimonadetes clearly clustered within the Proteobacteria reflecting the event of horizontal gene transfer that led to the acquisition of phototrophy in this group [10].

## Discussion

The phylogeny of BchF rules out the possibility that bacteriochlorophyll *a* synthesis originated within the phylum Chlorobi, Chloroflexi, Acidobacteria, or Proteobacteria. This is because the evolutionary event that led to the divergence of forms **A** and **B** must predate the diversification of the inner branches within both **A** and **B**, respectively. Thus, BchF, and therefore bacteriochlorophyll *a* specifically, most likely evolved for the first time in an ancestral phototrophic bacterium that predated the radiation of the current described phyla with strains that make bacteriochlorophyll *a*. Because, the origin of BchF is meaningless without chlorophyllide *a* oxidoreducatse (BchXYZ), it can be concluded that BchF originated in a bacterium that already had this enzyme and thus was capable of reducing chlorophyllide *a* to a form of bacteriochlorophyll; not unlike Heliobacteria today, for example.

The trees in **Fig 1** show that the BchF in Acidobacteria and Proteobacteria are closely related: however, the BchF in Acidobacteria is distinct to that in Proteobacteria, branching **before** the diversification of the proteobacterial forms. This close evolutionary relationship is also observed in other proteins of the chlorophyll synthesis pathway, including the set of proteins that make magnesium chelatase (BchDHI), protochlorophyllide *a* oxidoreductase (BchBLN/ChlBLN), chlorophyllide *a* oxidoreductase (BchXYZ), among several others [4, 8]. Moreover, this close evolutionary relationship of Acidobacteria and Proteobacteria is also reflected in the phylogeny of other proteins not involved in phototrophy [11, 12] and in every phylogenomic study produced in the past decade. In these studies, Acidobacteria cluster either as sister group of the class Deltaproteobacteria [13–18] or with Proteobacteria as the closest phylum containing phototrophic strains [19–21]. It can be deduced that the evolution of bacteriochlorophyll synthesis in Acidobacteria and Proteobacteria, including BchF, is more consistent with a vertical pattern of descent than with horizontal gene transfer and therefore the last common ancestor of these two groups was very likely phototrophic. This conclusion holds true whether Acidobacteria cluster as sister group of the class Deltaproteobacteria or not; and it is a strong indication that loss of phototrophy has occurred throughout both Acidobacteria and Protobacteria.

**Fig 2:**
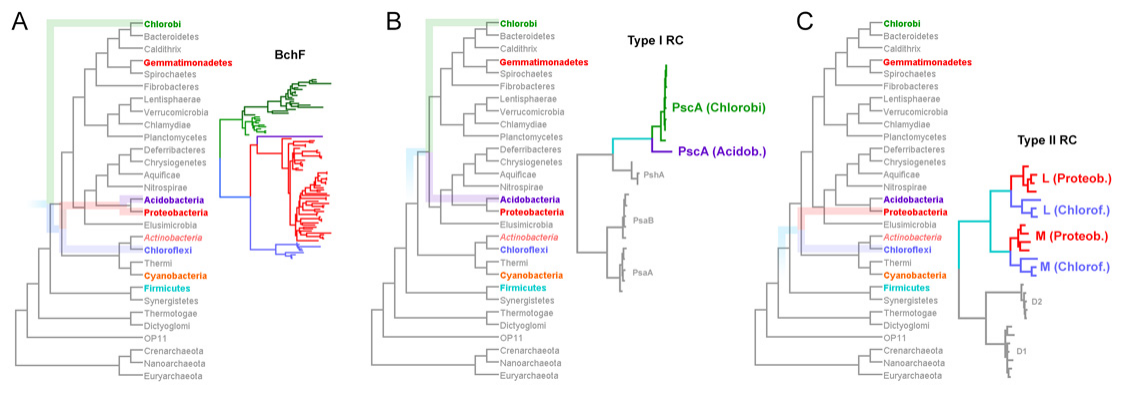
Evolution of bacteria in relation to BchF and reaction center proteins. **A** shows a phylogenetic tree of BchF overlaid over a phylogenomic tree of prokaryotes. B and C show those of Type I and Type II reaction center proteins over the phylogenomic tree, respectively. The phylogenomic tree that depicts the evolutionary relationship of different phyla of bacteria was obtained from Segata et al. [17], which was published open access. It was constructed using hundreds of proteins and thousands of genomes and the phylogenetic method, sequence alignments, and the phylogenetic tree are freely available from the author’s website (http://huttenhower.sph.harvard.edu/phylophlan). It is overall very similar to other phylogenomic trees, and the relationship of phyla containing phototrophic strains is virtually identical to those presented before, see for example Jun et al. [20], who implemented an alignment free approach. The phyla of bacteria highlighted in color represent those with photochemical reaction centers, with the exception of Actinobacteria that only recently was suggested to have been ancestrally phototrophic [2]. The colored transparent lines that are overlaid on top of the phylogenomic tree represent the evolution of the selected proteins. The phylogenetic relations of Type I (**B**) and Type II (**C**) reaction centers are well-established and were recently reviewed in detail [1].

*Chloracidobacterium thermophilum* has a homodimeric Type I reaction center [22]. Phylogenetically, this reaction center is more closely related to that found in the phylum Chlorobi than to those found in Heliobacteria and in the phylum Cyanobacteria [1]. It should be noted that the PscA protein of *Chloracidobacterium thermophilum* is different to those found in the phylum Chlorobi. As a result, the phylogenetic analysis of Type I reaction centers showed that the PscA in Acidobacteria branched out **before** the diversification of all known PscA within the phylum Chlorobi, see **Fig 2B** [1, 22]. This observation is conspicuous from the reaction center sequence data, which predict unique characteristics and structural variations for the PscA in Acidobacteria unlike those found in other groups. Based on current phylogenomic studies, the closest phylum to Acidobacteria containing strains with Type I reaction centers is—not surprisingly—the phylum Chlorobi [14, 16, 17, 19, 20] (**Fig 2**). As a result, the phylogenetic placement of the Type I reaction center protein from Acidobacteria and Chlorobi is consistent with the overall evolution of bacteria too. From this it follows that the phylum Chlorobi and the phylum Acidobacteria, including Proteobacteria, may have also shared a common phototrophic ancestor having a Type I reaction center. Because these phyla are separated by a number of non-phototrophic groups, it can be concluded that lack of phototrophy is indeed a derived trait.

Phototrophic members of the phyla Proteobacteria and Chloroflexi are characterized by having anoxygenic Type II reaction centers. The L and M core reaction center subunits found in Chloroflexi and Proteobacteria are more closely related to each other than they are to those found in cyanobacterial Photosystem II (D1 and D2). However, the L and M found in Chloroflexi are phylogenetically distinct to those found in Proteobacteria, thus forming separate clades (**Fig 2C**). Accordingly, anoxygenic Type II reaction center proteins (L and M) originated from an ancestral anoxygenic Type II reaction center protein that predated the divergence of the Chloroflexi-type and Proteobacteria-type L and M forms. This is independent of whether one phylum obtained Type II reaction center proteins by vertical descent or via horizontal gene transfer. This was first noted by Beanland [23] and was recently reviewed and discussed in detail [1]. The phylogenetic trees in **Fig 1** show that the BchF in Chloroflexi is more closely related to that in Proteobacteria than to that in the Chlorobi, and therefore it is consistent with both having bacteriochlorophyll *a* containing anoxygenic Type II reaction centers (**Fig 2C**) descending from an ancestral bacterium that predated the diversification of both phyla. Again, this is valid even if horizontal gene transfer occurred between their ancestors at some point in time.

The phylogenetic analysis presented in here suggests that the ancestral form of BchF originated early during the evolution of bacteria at a point in time that predated the diversification of the major groups of anoxygenic phototrophs. This raises several important questions on the early evolution of photosynthesis: could BchF and the bacteriochlorophyll *a* pigment have evolved before the last common ancestor to all phototrophic bacteria at the dawn of photosynthesis? Did the ancestors of Heliobacteria diverge at a point in time that predate the origin of bacteriochlorophyll *a*? Or did those ancestors lose BchF and BchC as a secondary adaptation to evolve bacteriochlorophyll *g*? Was the lineage of phototrophic bacteria that gave rise to the phylum Cyanobacteria able to synthetize any form of bacteriochlorophyll at all, or was this lineage never able to use bacteriochlorophyll since the origin of the first reaction centers?

Before attempting to answer these questions, it is necessary to understand the position of Cyanobacteria and Heliobacteria in the context of the evolution of bacteria and reaction centers too. The phylogenomic tree shown in **Fig 2** [17] shows that Cyanobacteria is in a supergroup that also contains the phyla Chloroflexi, Actinobacteria, and Thermi (*Deinochoccus*-*Thermus*). This phylogenetic relationship is somewhat well-established and has been reproduced in many independent studies implementing different methodologies [13, 14, 16, 17, 19–21]. It has been suggested that this supergroup originated early in the evolution of bacteria as it specialized to live on land [19]. Consistent with this, recent studies have shown that the phylum Cyanobacteria probably evolved on land and/or fresh water environments and only colonized the oceans during the end of the Proterozoic and early Phanerozoic periods [24, 25]. Because of these adaptations to land habitats, Battistuzzi et al. called this supergroup Terrabacteria; and the supergroup that contained Proteobacteria, Acidobacteria, Chlorobi, and closely related non-phototrophic groups, Hydrobacteria [19]. Taking into account that both Chloroflexi and Cyanobacteria are capable of photosynthesis, and adding to this the possibility that Actinobacteria was ancestrally capable of photosynthesis [2], it starts to look increasingly likely that this supergroup was also ancestrally phototrophic. There is a common unifying characteristic between these two supergroups: Type I and Type II reaction centers are found in both of them.

Now let us briefly consider the evolution of reaction centers proteins removed from the context of the evolution of bacteria; I have discussed this in more detail in a recent review [1]. The evolution of reaction centers can be viewed as the evolution of two proteins: a Type I and a Type II protein. Type I reaction center proteins, PscA, PshA, PsaA, and PsaB, are basically the same protein. They share significantly more sequence, structural, and functional homology with each other than with any Type II protein. This means that they originated from a common ancestral Type I reaction center protein. The differences among them are due to sequence divergence, which has increased with time as the different phyla of phototrophic bacteria diversified and specialized. Similarly, Type II reaction center proteins, L, M, D1, and D2, are the same protein; they share significantly more sequence, structural, and functional homology with each other than with any Type I protein. In other words, all Type II reaction center proteins descended from a common ancestral Type II protein. Yet, both Type I and Type II reaction center proteins share a common origin: that is to say, they originated from a common ancestral reaction center protein. It follows then that one of the earliest events in the history of photosynthesis is the divergence of Type I and Type II reaction center proteins, see for example [1, 26–28]. The fundamental consequence of this is that the evolutionary event that led to the divergence of Type I and Type II reaction center proteins should predate the diversification of each respective type itself. This is important because it implies that the origin of photosynthesis and two reaction centers should then predate the radiation of extant groups of phototrophic bacteria. If we use the phylogenomic tree in **Fig 2** as an example [17], then the origin of the enzymatic complexes required for the synthesis of bacteriochlorophyll, from Mg chelatase to chlorophyllide *a* oxidoreductase, the appearance of the first reaction center proteins, and the divergence of Type I and Type II reaction centers should have all occurred, at the very least, before the branching of the phylum Firmicutes. This is valid even if horizontal gene transfer of reaction center proteins occurred at any point in time.

If we assume that recent phylogenomic analyses are now converging toward the most accurate resolution of the bacterial tree of life possible with current techniques; then, based on the phylogeny in **Fig 1**, the ancestral BchF protein can be traced back to the point right before the split of the so-called Terrabacteria (**Fig 2**). If BchF emerged at this point, it implies that bacteriochlorophyll *a* should have originated after the divergence of Type I and Type II reaction centers, because as mentioned above, it is likely that at this stage in the evolution of photosynthesis, both reaction centers had already diverged and specialized. This in turn suggests that the ancestors to the phylum Cyanobacteria may have lost the capacity to make bacteriochlorophyll *a*, perhaps as an adaptation that was useful on the path to the emergence of oxygenic photosynthesis. In fact, the phylogeny of protochlorophyllide *a* oxidoreductase and chlorophyllide *a* oxidoreductase, as presented by Bryant et al. [4], is consistent with the cyanobacterial ancestors losing the latter enzyme at some point in time. However, this observation requires additional in-depth phylogenetic analyses to be verified with greater certainty.

In the case of Heliobacteria, recent phylogenomic analyses have placed the phylum Firmicutes as a clade branching out before all other phyla containing phototrophic bacteria, as seen in **Fig 2** for example, but see also [13, 16, 17, 20, 29, 30]. However, a couple of studies have suggested that the phylum Firmicutes clusters within the Terrabacteria instead [19, 21]. If the former hypothesis is correct, this would imply that Heliobacteria might have descended from a line of early evolving phototrophic Firmicutes that predated the evolution of the bacteriochlorophyll *a* pigment. On the contrary, if Firmicutes make part of the Terrabacteria then like Cyanobacteria, they should have descended from an ancestor that had BchF. Nevertheless, both possibilities are consistent with bacteriochlorophyll *a* evolving after the divergence of Type I and Type II reaction centers, because the presence of heliobacterial PshA-like reaction center proteins implies diversification of Type I reaction center proteins, and consequently the existence of Type II reaction center proteins as well.

In conclusion, the capacity to synthesize bacteriochlorophyll *a* originated only once in a phototrophic bacterium that predated―at the very least―the radiation event that gave rise to the phylum Chloroflexi, Chlorobi, Acidobacteria, and Proteobacteria. This places bacteriochlorophyll *a* synthesis at an early stage during the evolution of bacteria and implies that phototrophy might have been a common trait in ancestral populations of bacteria during the Archean; before the Great Oxygenation Event and before the rise and spread of eukaryotes. At the same time, the evolution of BchF suggests that the use of bacteriochlorophyll *a* pigment in photochemistry was possibly a late evolutionary innovation relative to the origin of photosynthesis, appearing only after the divergence and specialization of Type I and Type II reaction centers.

## Materials and Methods

Sequences were retrieved from the RefSeq database using the BchF protein from *Gemmatimonas phototrophica* as a query. A first BLAST was implemented restricted to the phylum Proteobacteria, and excluding environmental samples. A total of 286 BchF sequences were retrieved from Proteobacteria; however, only 50 sequences were used for the phylogenetic analysis as they all appeared to be highly similar and to have a monophyletic origin (see **S1 Fig**). A second BLAST was performed excluding Proteobacteria, which then retrieved all available sequences from the phylum Acidobacteria, Chlorobi, and Chloroflexi. No sequences belonging to members of the phyla Cyanobacteria and Firmicutes were found in the database. However, two sequences belonging to two different strains of the phylum Actinobacteria were retrieved: namely, *Asanoa ferruginea* and *Streptomyces purpureigenoscleroticus*. These two sequences had higher homology to strains in the class Alphaproteobacteria and whether these represent cases of horizontal gene transfer or genome contamination during sequencing was not determined in this work. Draft genomes of these two strains were deposited in the RefSeq database only recently (September, 2015) and there is not yet a publication associated with them.

Sequence alignments were performed using Clustal Omega employing ten combined guide tree and Hidden Markov Model iterations [31]. Maximum Likelihood phylogenetic analysis was performed using PhyML 3.1 [32] using the Blosum62 model of amino acid substitution. Amino acid equilibrium frequencies, proportion of invariable sites, and across site rate variation were allowed to be determined by the software from the dataset. Tree search operations were set as the best from the Nearest Neighbor Interchange and Subtree Pruning and Regrafting method, with the tree topology optimized using five random starts. Branch support was calculated with the Avarage Likelihood Ratio Test. Bayesian Inference was performed using Phylobayes 3.3f [33] using the CAT + GTR + Γ model and applying four discrete categories for the gamma distribution. Four chains were run until convergence. Trees were plotted using Dendroscope 3.4.1 [34].

## Supporting Information

S1 Dataset Phylogenetic trees and sequence alignments. The phylogenetic trees depicted in Fig 1 are provided in the Newick format. The sequence alignments for the BchF enzyme used in this work are provided in the Phylip format.
(ZIP)

S1 Fig Maximum Likelihood phylogenetic tree of BchF including all proteobacterial sequences. The tree shows that all proteobacterial sequences are highly similar and are monophyletic. The tree was constructed using the same conditions as described in Materials and Methods.
(TIF)
