# Supplementary figures and images for: Origin of Bacteriochlorophyll a and the Early Diversification of Photosynthesis
Source: PLoS One. 2016 Mar 8;11(3):e0151250. doi: 10.1371/journal.pone.0151250 (PMC4783071; doi:10.1371/journal.pone.0151250)

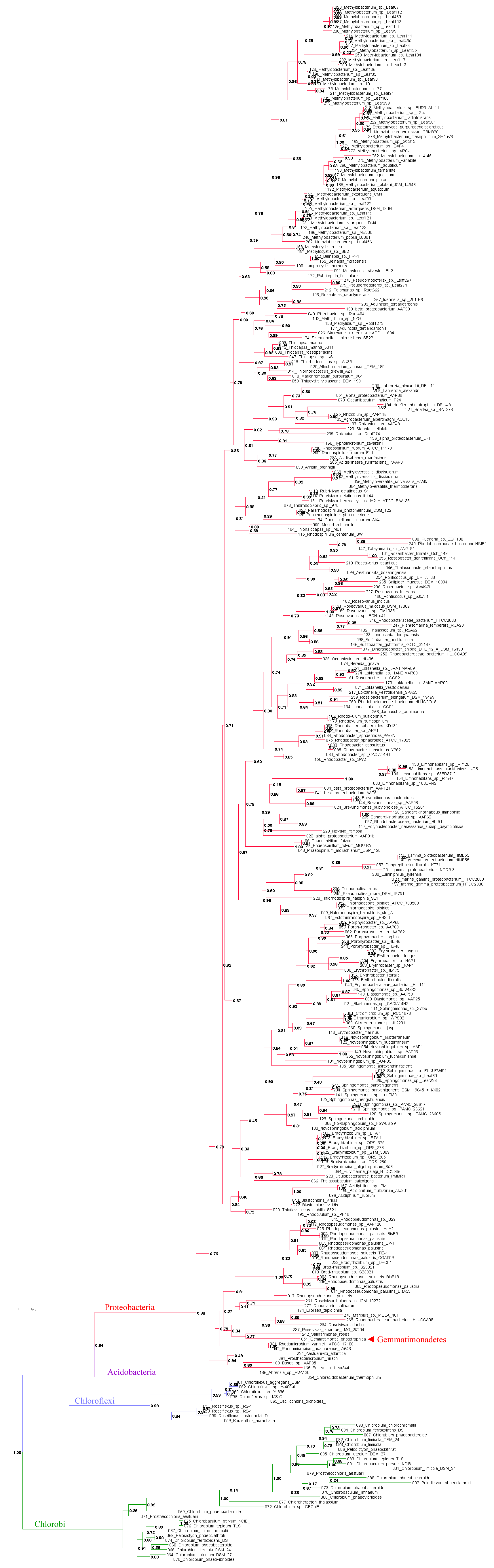

Supplement: S1 Fig — The tree shows that all proteobacterial sequences are highly similar and are monophyletic. The tree was constructed using the same conditions as described in Materials and Methods. (TIF) [file pone.0151250.s002.tif]
